# Systematic evaluation of COVID-19 related Internet health rumors during the breaking out period of COVID-19 in China

**DOI:** 10.34172/hpp.2021.37

**Published:** 2021-08-18

**Authors:** Ge Pu, Liu Jin, Han Xiao, Wei Shu-ting, He Xi-zhe, Tang Ying, Xu Xin, Wang Sheng-yuan, Bian Ying, Wu Yibo

**Affiliations:** ^1^Institute of Chinese Medical Sciences & State Key Laboratory of Quality Research in Chinese Medicine, University of Macau, Avenida da Universidade, Taipa, Macau 999078, China; ^2^The Third Clinical Department, China Medical University,Shenyang 110013,Liaoning Province,China; ^3^School of Pharmaceutical Sciences, Sun Yat-Sen University, Guangzhou 510006, Guangdong Province, China; ^4^Cheeloo College of Medicine,ShanDong University,Jinan 250012,Shandong Province,China; ^5^Jiangxi University of Traditional Chinese Medicine,Nanchang 330004,Jiangxi Province,China; ^6^Changzhi Medical College,Changzhi 046000,Shanxi Province,China; ^7^School of Life Science, Peking University, Beijing 100871,China; ^8^Liaoning Technical University College of the Media and Arts, Fuxin 123000,China; ^9^School of Public Health, Peking University, Beijing 100191,China; ^10^Key Research Base of Philosophy and Social Sciences in Shaanxi Province, Health Culture Research Center of Shaanxi, Xi’an 712046,China

**Keywords:** COVID-19, Health rumor, Systematic evaluation, China, The breaking out period of COVID-19

## Abstract

**Background:** To adapt the scientific evaluation tool for the confusion evaluation of health rumors and to test this tool to the confusion evaluation of coronavirus disease 2019 (COVID-19)-related health rumors on Chinese online platforms during the outbreak period of COVID-19in China.

** Methods:** The design of our study was systematic evaluation of COVID-19-related health rumors. Retrieved from 7 rumor-repellent platforms, rumors about COVID-19 were collected during the publication from December 1, 2019, to February 6, 2020, and their origins were traced. Researchers evaluated rumors using the confusion evaluation tool in 6 dimensions(creators, evidence selection, evidence evaluation, evidence application, backing and publication platform, conflict of interest). Items were scored using a seven-point Likert scale. The scores were converted into percentages, and the median of rumors from different sources was compared with rank-sum test.

**Results:** Our research included 127 rumors. Scores were converted to percentages, median and interquartile range are used to describe the data. The median score: creators 25.00%(interquartile range, IQR, 16.67-37.50%), evidence selection 27.78% (IQR, 13.89-44.44%),evidence evaluation 33.33% (IQR, 25.00-45.83%), evidence application 36.11% (IQR, 22.22-47.22%), backing and publication platform 8.33% (IQR, 4.17-20.83%), conflict of interest75.00% (IQR, 50.00-83.33%). Almost 40% rumors came from WeChat and the rumors with the lowest scores were concentrated on the WeChat platform. The rumors about prevention methods have relatively lower scores.

**Conclusion:** Most rumors included were not highly confusing for evaluators of this project.WeChat is the "worst-hit area" of COVID-19 related health rumors. More than half rumors focus on the description of prevention methods, which reflects the panic, anxiety and blind conformity of the public under public health emergencies.

## Introduction

The outbreak of coronavirus disease 2019 (COVID-19) epidemic occurred in mid-December 2019. The COVID-19 pandemic has not only caused significant challenges for health system all over the globe but also fueled the surge of numerous rumors, hoaxes and misinformation, regarding etiology, outcomes, prevention, and cure of the disease.^1^ The social media panic traveled faster than the COVID-19 spread. For example, someone claimed on the Internet that chloroquine can prevent and treat COVID-19. Cases of hospitalization occurred due to overdose of chloroquine to prevent COVID-19 worldwide.

WHO Director General Dr. Tedros called this the fight against “trolls and conspiracy theories”. Misinformation causes confusion and spreads fear, thereby hampering the response to the outbreak. “Misinformation on the coronavirus might be the most contagious thing about it”, he said. Rumors and misinformation regarding remedies and cures led to panic buying during the outbreak, and timely clarification of rumors effectively reduced irrational behaviour.^2^

In addition to addressing the urgent need to scale-up public health measures to combat the outbreak, we need to combat the pandemic of social media panic. To this end, it is important to conduct spatiotemporal analyses of the discourse and its association, or disassociation, with the epidemiological situation as this will allow spatiotemporal targeted communication and intervention campaigns to be executed by public health authorities. We need to rapidly detect and respond to public rumors, perceptions, attitudes and behaviors around COVID-19 and control measures.

It is found that the public pays attention to the epidemic situation and has certain cognition of it, but for them there is a blind area of knowledge for false information on the network, which leads to a serious misunderstanding, so the relevant knowledge level needs to be improved.^3^ The characteristics of decentralization, anonymity, and low threshold of Internet communication provide culture media for the spread of health rumors. These rumors may disturb the normal order of social economy, affect people’s normal life, and may even reduce the credibility of the country and affect the governing ability.^4,5^

At present, many rumor-removal platforms have been established in China, such as China Internet Joint Rumor-Removal Platform and Baidu Rumor-Removal Platform. There are few summaries and systematic reviews on network health information. Drawing on evidence-based ideas, Pan et al put forward the concept of evidence-based science popularization, that is, “prudently, accurately and wisely apply the best research evidence obtained, combining the professional knowledge of popular science workers, considering the needs and health literacy, so as to create scientifically readable quality popular science works”.^6^ Based on evidence-based thinking, this study intends to conduct a systematic evaluation of COVID-19 health rumors according to the six dimensions of creators of information, evidence selection, evidence evaluation, application of evidence, backing and publishing platform, and conflict of interest. The evaluation tool we used is Preliminary Expert Consensus on the Scientific Evaluation Standard of Health Works for Public which is formed by three rounds of Delphi expert consultation on the basis of AGREE II method and applies to health information which is presented and disseminated in a way that is easy for the public to understand, accept, and participate.

## Materials and Methods

This study was performed in accordance with Preferred Reporting Items for Systematic Reviews and Meta-Analysis (PRISMA).^7^ COVID-19-related health rumors on Chinese online platforms were considered and our search was initiated on December 1, 2019 until February 6, 2020.

### Data sources

Rumors about COVID-19 in the present study from seven rumor-repellent platforms, including Scientific Rumor-Repellent Platform (https://piyao.kepuchina.cn), China Internet Joint Rumor Platform (http://www.piyao.org.cn), Tadpole Stave Rumor-Repellent Platform (http://news.kedo.gov.cn/kxpy), Dingxiangyuan COVID-19 Rumor-Repellent Platform (https://ncov.dxy.cn/ncovh5/view/pneumonia), Today’s Headline Epidemic Prevention and Control Rumor Area (http://www.piyao.org.cn/2020yqpy), Tencent ’s Verification Platform (https://vp.fact.qq.com/home) and Baidu Rumor-Repellent Platform (https://mbd.baidu.com/newspage/data/mdpage?tag=8&id=5807).

### Selection criteria

All rumors published in seven platforms were included. The publication time was limited from December 1, 2019 to February 6, 2020. All the rumors not related to 2019-nCoV were excluded. And rumors whose authenticity cannot be distinguished or source cannot be found or have been published repeatedly were excluded.

### Search methods

For the rumor platforms with rumor refutation section, all rumors the conclusion of which are “false” were included. For the rumor platforms that need to search the keywords, we searched the keywords “pneumonia, coronavirus, COVID-19” and included all rumors the conclusion of which are “false”. Two investigators independently searched the rumors from seven platforms and another investigator checked and decided on inclusion.

Considering the heterogeneity in different rumor-repellent platforms with regard to the judgement criteria to identify rumors, a second screening of rumors was conducted for the purpose of confirming whether the rumors meet the consistent definition.

**Figure 1:**
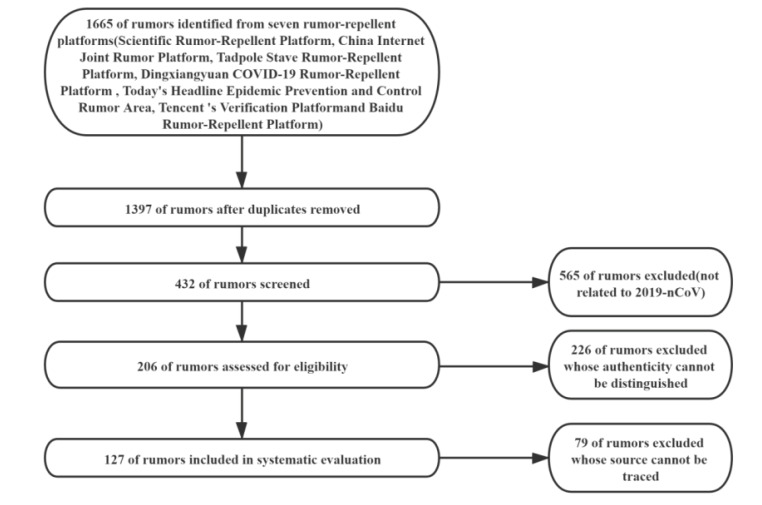
Selection of rumors included in the evaluation

Tracing the source of rumors: Use searching tools such as Baidu search, Sogou search, WeChat search, etc. to conduct a synonymous expression search for rumors that met the inclusion criteria, so as to find the source of the rumors. Use split search for rumors containing multiple content. The process of rumor selection is shown in Figure 1.

### Rumor evaluation

Researchers who had finished training conducted by the scientific evaluation standard for health science popularization expert consensus formulation team used the evaluation tool of Preliminary Expert Consensus on the Scientific Evaluation Standard of Health information for Public to independently score the included websites or articles of rumor sources from 14 items in 6 dimensions (creators of information, evidence selection, evidence evaluation, evidence application, backing and publication platform, conflict of interest) (Read supplementary materials for details). The tool is shown in Appendix 1. Before the evaluation, two researchers carefully studied COVID-19 treatment plan (Trial Seventh Edition). Before each evaluation, two researchers communicated with each other to reduce subjectivity. All items were scored according to the Likert 7 scale, and 1-7 points in turn represents strongly disagree, disagree, relatively disagree, general, relatively agree, agree and strongly agree. If the difference between 2 researchers on an item was greater than or equal to 3 points, 2 researchers would seek consensus first, and if the consensus was not reached, the third researcher would join in the discussion to reach the final conclusion. If no consensus was reached, members in the scientific evaluation standard for health science popularization expert consensus formulation team would be invited to participate in the discussion. If the rumor source did not quote expert opinions, item 8 (Expert opinions without high-level evidence should be carefully quoted in health information) of dimension 3 (evidence evaluation) was rated as “not applicable”, and the rumor whose item 8 was counted as “not applicable” would not count evidence evaluation score.^8^

**Appendix 1:** Rumor Systematic Evaluation Tool

**First level entries**	**No.**	**Secondary entries**	**Score**
Creators	1	Authors of health information should have a professional background in the fields involved in the information and a full understanding of the fields discussed.	
	2	Authors of health information should not only have the experience of science popularization writing, but also have a strong ability to transform professional knowledge into science popularization information and retain its scientific nature.	
Evidence selection	3	In the selection of health science information, the viewpoints related to the issues discussed should be fully presented and the selection bias should be avoided as far as possible.	
	4	When the core conclusion of health information is discussed, it should be demonstrated with high-level evidence.	
	5	Health information should list the sources of evidence cited.	
Evidence evaluation	6	Controversial evidence should be avoided in health information as much as possible.	
	7	Evidence for health information should be representative examples and avoid special cases to represent the general situation to prove the author’s views.	
	8	Expert opinions without high-level evidence should be carefully quoted in health information	
Application of evidence	9	The problem of the definition of a health science work should be scientific. At the same time, the evidence of information selection should be able to prove the core issues raised.	
	10	The conclusion of a work should have sufficient evidence, and the process of proving the conclusion should be fully discussed through proper logical proof	
	11	When the article comes to a conclusion and forms a recommendation, it should weigh the advantages and disadvantages comprehensively and consider whether the recommendation has other risks, operability and necessity	
Backing and publishing platform	12	Health information shall be supported by professional scholars or organizations in relevant fields.	
	13	Health information published on authoritative platforms are generally more scientific.	
Conflict of interest	14	Health information should not violate the principle of conflict of interest	

### Data analysis

The score of each dimension was equal to the sum of the scores of each item of each reviewer in that dimension, and was standardized as the percentage of the highest possible score of this dimension. The scoring method refers to AGREE II (The Appraisal of Guidelines for Research and Evaluation).^9^

Maximum possible score = Maximum score of the 7-level scale × the number of dimension items × the number of reviewers

Minimum possible score = Minimum score of the 7-level scale × the number of dimension items × the number of reviewers

The calculation score of each dimension according to the scoring formula is:

$$Score\;of\;each\;\dim ension=\;\frac{Obtained\;score-Minimum\;posssible\;score}{Maximum\;possible\;score-Minimum\;possible\;score}$$

Rumors with scores greater than or equal to 50% in all 6 dimensions were considered highly confusing, and rumors with scores less than 50% in all 6 dimensions were considered less confusing. In each dimension, the higher the score of the rumor, the more confusing it is.

All data was analyzed with IBM SPSS Statistics 25.0 (Network Version from Peking University, Address: 162.135.134.153). The scores of health rumors did not conform to the normal distribution, so the median and interquartile range were used to represent the central and discrete trends. The influence of different sources, different classifications and different conclusions of refutation on the scores of health rumors was analysed by Wilcoxon sum test. The intra-class correlation coefficient (ICC) and the two-way random effect model were used to evaluate the consistency between the two reviewers. The correlation strength of the coefficient was evaluated as: poor (<0.40), moderate (0.40-0.59), good (0.60-0.74) or excellent (0.75-1.00).^10^ Take *P*< 0.05 as the standard of statistical test difference.

## Results

### Search results of rumors and basic characteristics of rumors included

Our research included a total of 127 rumors. The rumors are divided into 9 categories: virological characteristics, transmission channels, infectious sources, disease prognosis, modern medicine treatment, susceptible population, prevention methods, symptoms and diagnosis, and traditional Chinese medicine treatment. The sources of rumors are mainly divided into 8 categories: Baidu Tieba, Sohuhao, WeChat Public Account, WeChat Moments, WeChat Group, Sina Weibo, Zhihu, other websites and applications (such as bilibili barrage video network, Tiktok, etc).

### Rumor evaluation results

The basic information of ten representative rumors with the highest and lowest scores in each dimension is on Table 1.

**Table 1:** The basic information of ten representative rumors with the highest/lowest scores in several dimensions

**No.**	**Rumor content**	**Source**	**Classification**	**Creators**	**Evidence selection**	**Evidence evaluation**	**Application of evidence**	**Backing and publishing platform**	**Conflict of interest**
1	The mysterious virus has been confirmed as a new SARS virus.	People's website	Virological characteristics	79 (highest)	83 (highest)	75 (highest)	50	42	100 (highest)
2	The 2019-nCoV can be suspended in the air for one day.	Baidu Tieba Guangrao	Virological characteristics	0 (lowest)	8	Not applicable	36	4	100 (highest)
3	Snow can kill the 2019-nCoV.	Baidu Tieba Wuhan	Virological characteristics	4	0 (lowest)	Not applicable	6	4	92
4	The 2019-nCoV is the evolution of the SARS virus.	Baidu Tieba 2019-nCoV	Virological characteristics	8	6	Not applicable	19	0 (lowest)	67
5	Eye contact can spread COVID-19	WeChat Group	way for spreading	0 (lowest)	0 (lowest)	Not applicable	3	0 (lowest)	33
6	There is evidence that mosquitoes and flies can spread the COVID-19	Baidu Tieba 20(lowest)19-nCoV	way for spreading	0 (lowest)	0 (lowest)	Not applicable	8	4	58
7	Children and adolescents are not susceptible to the 2019-nCoV.	Sanxiang Metropolis Daily Baijia Hao	Susceptible population	33	33	22	28	25	100 (highest)
8	Professor Zhong Nanshan said vegetarians will not be infected with the 2019-nCoV.	Vegetarian Marketing Network	Susceptible population	25	19	17	11	8	0 (lowest)
9	Oseltamivir and antibacterial drugs can prevent COVID-19.	China Economic Net	Prevention method	63	64	56	58	71 (highest)	92
10	Smoking can prevent 2019-nCoV infection.	WeChat Moments	Prevention method	4	6	Not applicable	11	0 (lowest)	50

One of the included rumors scores greater than or equal to 50% in six major dimensions, namely “Oseltavir and antibacterial drugs can prevent COVID-19” of China Economic Net. This rumor is highly confusing. Other rumors included are not highly confusing, which account for 99.21% of all included rumors.

There are 15 rumors scoring less than 50% in 6 major dimensions. Two of the 15 rumors are originated from WeChat group, which are as follows: “Alcohol of high degree can resist COVID-19”, “Dancon tea and strawberry can prevent COVID-19”. Six of the 15 rumors are originated from WeChat Public Account, which are as follows: “Virus protection card can kill COVID-19 virus”, “Probiotics can prevent COVID-19”, “Dettol, Welsh and other household hand sanitizers can kill COVID-19 virus”, “People should wear goggles to prevent the COVID-19 from invading the body”, “Saline cleaning the nose can prevent the COVID-19” and “Betel nut can resist the COVID-19”. Two of the 15 rumors are originated from Baidu Tieba, which are as follows: “COVID-19 has been mixed in the air, and it will infect you once you breathe” and “Aspirin can delay the course of COVID-19”. Two of the 15 rumors are originated from Zhihu, which are as follows: “The steam sprayed from the ironing machine can be used for clothes disinfection” and “Eating chili can reduce the risk of death caused by COVID-19”. Other rumors include “Vegetarians are not infected with the COVID-19” from the vegetarian marketing network, “Drinking can resist COVID-19 virus” from the WeChat Circle of Friends and “Families who keep pets are not infected with COVID-19” from Sina Weibo.

In addition, of the 58 rumors that are not scored in the “evidence evaluation” dimension, 7 scored less than 50% in the remaining 5 dimensions. Among these rumors, 3 are originated from the WeChat group, which are “Eyesight can spread COVID-19”, “The whole body pain and hemiplegia will occur in the patients who have been cured of COVID-19” and “The sow says that eating 9 eggs can prevent COVID-19”. Two of the 7 rumors are originated from Zhihu, which are “Drinking boy urine can prevent COVID-19” and “If used masks are put in a disinfection cabinet to be sterilized, they can continue to be used”. Two of the 7 rumors are “COVID-19 was leaked from Wuhan” from Twitter and “Masks can be sterilized using microwave oven” from Wanwei Home Grid Home Appliance Encyclopedia.

These 22 rumors are less confusing and easily recognized. The distribution, the median and the results of the consistency test between the scorers (the consistency is good, ICC is greater than or equal to 0.75) of rumors in each dimension can be seen in Table 2.

**Table 2:** Distribution and median of rumors in each dimension and the consistency test between two raters

**Dimension**		**Scores≥50%**	**Scores<50%**	**Median% (Q1-Q3)**	**ICC (95% CI** ^*^ **)**
		**N**	**N**		
1	Creators	17	110	25.00 (16.67-37.50)	0.740 (0.650-0.810)
2	Evidence selection	25	102	27.78 (13.89-44.44)	0.856 (0.801-0.896)
3	Evidence evaluation	12	57^a^	33.33 (25.00-45.83)^c^	0.726 (0.591-0.821)^c^
4	Application of evidence	27	100	36.11 (22.22-47.22)	0.712 (0.614-0.788)
5	Backing and publishing platform	1	126	8.33 (4.17-20.83)	0.779 (0.700-0.839)
6	Conflict of interest	103^b^	24	75.00 (50.00-83.33)	0.694(0.591-0.774)

* 95%CI means 95% confidence intervals ^a^ There are 58 rumors that did not quote expert opinions, and those which are recognized as “not applicable” at the time of scoring would not be counted for this dimension. ^b^ There are 6 rumors that the dimension score is 100%. ^c^ Rumors which are recognized as “not applicable” in item 8 (Expert opinions without high-level evidence should be carefully quoted in health works) of dimension 3 would not be counted in this dimension.

### Comparison of rumor scores from different sources

Among the rumors from different sources, 50 rumors are originated from the WeChat (WeChat public account, WeChat circle of friends, WeChat group), accounting for almost 40% of all platforms.

In terms of the median score of each dimension, the source with lowest scores of creators, evidence selection, evidence application, backing and publishing platform is WeChat group, the source with lowest scores of conflict of interest is WeChat circle of friends. In the dimensions of evidence evaluation, there is no statistical difference between rumors from different sources (*P*≥0.05), and the rumors with the lowest scores in each dimension are concentrated on the WeChat platform. The results above can be seen in Table 3.

**Table 3:** Comparison of rumor scores from different sources by rank sum test

**Sources**	**n**	**Scores of each dimension(%)**					
		**Creators**	**Evidence selection**	**Evidence evaluation** ^*^	**Application of evidence**	**Peer review and publishing platform**	**Conflict of interest**
A	12	12.50 (1.04.-23.96)	12.50 (5.56-26.39)	29.17 (25.70-45.83)	33.34 (9.72-44.44)	4.17 (4.17-7.29)	75.00 (60.42-89.59)
B	7	29.17 (20.83-41.67)	36.11 (22.22-61.11)	38.89 (27.78-50.00)	52.78 (44.44-52.78)	25.00 (4.17-29.17)	83.33 (83.33-83.33)
C	31	29.17 (20.83-45.83)	33.33 (25.00-47.22)	38.8925.00-47.22)	44.44 (30.56-50.00)	12.50 (8.33-20.83)	75.00 (50.00-83.33)
D	5	20.83 (10.42-27.08)	16.67 (8.34-37.50)	23.61 (6.95-29.87)	27.78 (12.50-33.34)	8.33 (2.09-12.50)	50.00 (33.34-50.00)
E	14	2.09 (0.00-8.33)	8.33 (4.17-16.67)	2.78 (0.70-34.03)	12.50 (2.78-25.70)	2.09 (0.00-5.21)	58.34 (33.33-68.75)
F	5	16.67 (10.41-37.50)	41.67 (9.73-59.73)	41.67 (27.78-66.67)	33.33 (19.45-50.00)	4.17 (4.17-27.09)	75.00 (41.67-91.67)
G	26	20.83 (16.67-31.25)	22.22 (13.89-44.44)	30.56 (27.78-41.67)	37.50 (29.17-42.36)	8.33 (8.33-12.50)	66.67 (50.00-75.00)
H	27	37.50 (25.00-50.00)	33.33 (22.22-44.44)	33.33 (25.00-50.00)	41.67 (30.56-52.78)	20.83 (8.33-29.17)	83.33 (66.67-91.67)
H (K)		47.631	29.333	10.534	30.334	43.168	21.478
*P*		0.000^a^	0.000^a^	0.160	0.000^a^	0.000^a^	0.003^a^
Paired comparison（P）		A-C (0.034) A-H (0.001) E-B (0.010) E-C (0.000) E-G (0.034) E-H (0.000)	E-B (0.032) E-C (0.001) E-H (0.001)		E-B (0.002) E-C (0.000) E-H (0.002)	A-C (0.008) A-H (0.001) E-C (0.000) E-H (0.000) G-H (0.045)	D-B (0.045)

Annotation: A is Baidu Tieba, B is Sohu Hao, C is WeChat public account, D is WeChat Moments, E is WeChat group, F is Sina Weibo, G is Zhihu and H is others. * Rumors which are recognized as “not applicable” in item 8 (Expert opinions without high-level evidence should be carefully quoted in health works) of dimension 3 would not be counted in this dimension. ^a^ *P* < 0.05 indicated significant difference.

### Comparison of rumor scores in different categories

Among the 77 (60.63%) rumors about COVID-19 prevention methods, one scores greater than or equal to 50% in all dimensions, and 15 (19.48%) score less than 50% in all dimensions (including that 4 of “evidence evaluation” dimension are not scored). Among the other 50 rumors, none scores greater than or equal to 50% in all dimensions, and 6(12%) score less than 50% in all dimensions (including one that “evidence evaluation” dimension is not scored).

The median score of each dimension of the rumors about prevention methods is less than other rumors. The results above can be seen in Table 4.

**Table 4:** Descriptive statistics of rumor scores from different categories

**Dimension**		**Preventive methods(77)**	**Others(50)**
		**Median%(Q1-Q3)**	**Median%(Q1-Q3)**
1	Creators	20.83(12.50-37.50)	25.00 (16.67-37.50)
2	Evidence selection	25.00 (13.89-41.67)	29.17 (13.89-52.78)
3	Evidence evaluation	30.56 (25.00-41.67)^*^	41.67 (27.78-50.00)^*^
4	Application of evidence	36.11 (22.22-47.22)	37.50 (24.31-50.00)
5	Peer review and publishing platform	8.33 (4.17-16.67)	10.42 (4.17-25.00)
6	Conflict of interest	66.67 (50.00-83.33)	75.00 (50.00-83.33)

* Rumors which are recognized as “not applicable” in item 8 (Expert opinions without high-level evidence should be carefully quoted in health works) of dimension 3 would not be counted in this dimension.

## Discussion

Many researchers have studied rumors about COVID-19 during the pandemic. Dong et al^11^ collected the netizen comments of “People’s Daily” on Sina Weibo, a popular social media platform in China, to extract potential emotional information, and collected rumor data through the Tencent Verification Platform to test the relationship between public sentiment and rumors Relationship. They found that rumors are a catalyst for public emotions. Disproving them in a timely manner would be helpful to increase positive emotions of the public. Fearful rumors were associated with fear. Thus, media platforms should strengthen the monitoring of online rumors, identify and verify emotional rumors in a timely manner, and minimize the spread of fearful rumors to reduce fear among the public.

Chen et al^12^ collected internet rumors through Sina Weibo platform. They divided the outbreak of COVID-19 into five periods, and compared the rumor classification, public focus and hot spots of the five periods. The study found that WeChat had become the main source of COVID-19 rumors, and there were significant differences in the form and source of rumors in five different periods, indicating the requirement to establish a rumor monitoring and refutation mechanism, release official information in time, and adjust policy at different periods.

Zhang et al^13^ used the interview method to study the causes of the rumors that “Shuanghuanglian oral liquid can COVID-19” and the reasons why people snapped up Shuanghuanglian oral liquid. They proposed three principles that can help effective health communication and health rumors management, which are maintaining the intelligibility of information, keeping the accuracy of information, enhancing the credibility of information. However, there is currently a lack of research in the world to evaluate the confusing nature of rumors related to the COVID-19. We have developed a tool for evaluating the confusing nature of rumors and used it to systematic evaluation of COVID-19 related Internet health rumors during the breaking out period of COVID-19 in China.

The popularization of health science is an important part of the Plan of Healthy China 2030. It is of great significance to establish the evaluation standard of health works for public in order to solve the problem of mixed quality in the field of health science popularization in China. The scientific evaluation of health science should be based on evidence-based method, according to six aspects: author, evidence selection, evidence application, evidence evaluation, peer review& publishing platform and conflict of interest. Preliminary Expert Consensus on the Scientific Evaluation Standard of Health Works for Public is published in February, 2020. As far as we know, this is the first expert consensus on the scientific evaluation standard of health works for public in China. Through consultation by nearly 100 experts from Delphi, it was jointly published by Youth Science Group of Popular Medical Writing Committee, China Science Writer Association and National Health Accomplishment Promotion Committee and China Health Culture Association.^8^ We have modified this tool and used it for the confusing evaluation of health rumors related to COVID-19.

Among the 127 rumors included in this study, the scores of 1 rumor are greater than or equal to 50% in all dimensions, which is Oseltamivir and antibacterial drugs can prevent COVID-19. The rumor originated from a piece of news from China Pharmaceutical University. On January 31, 2020, major Chinese media reported that China Pharmaceutical University adopted Ligand-based drug design and screened out more than a dozen drugs that can fight COVID-19, including oseltamivir, an anti-flu drug and 10 antibacterial drugs such as vancomycin, piperacillin, ampicillin, amikacin, azithromycin, moxifloxacin, etc. Since the public does not understand the specific process of drug development, they believe that the drugs screened in this way can fight COVID-19. The spread of this rumor is related to the low scientific literacy of the Chinese public, the blind conformity mentality during the epidemic and the inadequacy of the national emergency science popularization policy. In addition, in the early stage of the epidemic, some hospitals, such as the Third People’s Hospital of Hubei province affiliated to Jianghan University, tried to prescribe oseltamivir for patients with COVID-19. Some media reported the treatment plan.^14^ This may also be one of the reasons why this rumor was generated and spread. Zhou et al^15^designed a questionnaire to assess the health perceptions and misunderstanding about COVID-19 in different groups of people, indicating that the increase of information channels can improve the public’s health perceptions and help them identify rumors and other misleading information. Therefore, the government should strengthen the supervision of social media and improve the influence of expert opinions to reduce the risk of rumor spread. To prevent the public from misreading news reports, the media should add appropriate warnings after the report.

The scores of 15 rumors are less than 50% in all dimensions. Since the scores of each dimension do not conform to the normal distribution, the median is used to represent the overall situation. The medians of each dimension from largest to smallest are as follows: Conflict of interest(75.00%), application of evidence (36.11%), evidence evaluation (33.33%), evidence selection (27.78%), creators (25.00%) and backing and publishing platform (8.33%)

The rumor scores of choice of evidence, evidence evaluation and evidence application are generally low, of which the dimension of evidence selection is more prominent. When choosing evidence, many rumors chose evidence that was controversial and biased, lacked advanced evidence, and did not list the cited sources of evidence. It is worth noting that part of rumors citing expert opinion quoted the fictional opinions. These rumors are packaged with science, mixed with all kinds of information. Moreover, such rumors fully draw lessons from and make full use of the law of communication, and make good use of background information and scientific logic to support the point of view with a large number of data, schematic maps and citation of technical terms.^16,17^ Expect for 58 rumors not citing expert opinion, 56 of the remaining 69 rumors score less than 50% in the evidence evaluation dimension, which further proves that most rumors have the problem of generalizing in the selection and evaluation of evidence. The dimension of evidence application generally shows that the reliability of the defined problem is poor, the evidence and argumentation process are not enough to prove the question, and there is poor consideration in the process of forming a recommendation.

The dimension of backing and publication platform is the dimension with the lowest median, which indicates that most rumors are published on platforms that are unconvincing, and experts in related fields generally have good judgment and generally do not publish unconfirmed remarks.

The dimension of conflict of interest has the highest median, which reveals the psychological characteristics of the public in starting and spreading rumors. Only a small part of the public spread rumors for the purpose of profit. Most of the public spreading rumors are based on the following three types of psychology. The first one is the stress avoidance psychology of issue of “social risk”. Under the aggravating situation of the epidemic, people tend to ease their anxiety and fear by accepting and spreading rumors to vent the panic and dissatisfaction with the status, in order to make up for the psychological emptiness or seek sense of security. The second one is the psychology of self-realization under “use and satisfaction”, according to the “third person” hypothesis, some people “kindly” transfer them to needy friends when they come into contact with rumors. The third one is the mentality of following the crowd under “group consciousness”, the repeated emergence of a topic in a group is easy to induce individuals to accept and forward the content of the discussion without thinking.^18,19^

Among 127 rumors, 50 rumors originated from WeChat, which accounts for almost 39% of all rumors included. It indicates that WeChat is the “worst-hit area” of COVID-19 related health rumors spreading in China. WeChat, as an emerging Media platform, whose monthly active accounts exceeded 1.1 billion in 2019 according to the 2019 WeChat Annual Report, is gradually becoming an important platform of health information production and spreading due to the characteristics of low entry barrier, convenience of use, “decentralization” and so on.^20,21^ During the epidemic, rumors spread by WeChat come out one after the other. Health rumors mainly based on prevention method are widespread and score generally low. 2019 Network Rumor Governance Report pointed out that medical care and health was the high dimension of network rumors. For most people, it is difficult to distinguish the truth from falsehood only by their own knowledge reserve, however, severe situation clearly shows the threat to life and health, taking “prevention behavior” without thinking and farce caused by blind conformities.^22,23^

Based on the results, we make the following recommendations to the government, the media, social networking sites and the public.

First, the government should further popularize basic education, actively organize and carry out science popularization activities and improve the scientific literacy of the public so as to improve the ability of the public to identify rumors. Second, it should intensify the verification of the information content on the Internet, focus on WeChat and other platforms that generate more rumors and punish those that publish false information on the Internet and cause serious consequences. Third, it should intensify efforts to refute rumors, establish and improve the public opinion monitoring mechanism, work closely with relevant experts and media, popularize relevant knowledge and refute rumors in areas of public concern (such as the prevention methods of COVID-19) by means of press conferences.

As for the media and social networking sites, they should establish and improve the verification mechanism of scientificity of the content, pay attention to the verification of scientificity of published content. They should also actively cooperate with the work of refuting rumors and establish relevant columns, special programs or websites to be “vanguard soldiers” in refuting rumors.

As for the public, they should actively study scientific knowledge and improve scientific literacy. They should also use critical thinking to evaluate the credibility and quality of the content, verify the content in varies ways, keep alert to inaccurate information, and improve media literacy.

## Conclusion

Among the 127 COVID-19-related health rumors included in this study, the great majority of the rumors were not highly confusing for evaluators of this project. WeChat is the “worst-hit area” of COVID-19-related health rumors spreading in China, which accounts for almost 40% rumors included in this research. More than half of the COVID-19-related rumors included in this study focused on the description of COVID-19 prevention methods, which reflects the panic, anxiety and blind conformity of the public under public health emergencies. Our research has also explored a method that can be used for the confusion evaluation of health rumors, which deserves further study.

Due to the intensified efforts of refuting rumors on the Internet, some original pages of rumors have been deleted. When tracing the source of rumors, it is difficult to find the real source, which may result in incomplete information of the obtained rumors. As this article only analyzes rumors traceable on the seven platforms, a large number of rumors that are not traceable or not on the seven platforms have not been analyzed. In addition, there is heterogeneity in rumor-repellent platforms regarding the judgement criteria to identify rumors. Therefore, we conducted a second screening to minimize the differences between them. Finally, some rumors are presented in the form of videos or pictures, and the tools used in this study are mainly used to analyze the text of health science works, which has some problems in applicability. This study can only show that the included health rumors are not highly confusing for people who have a certain understanding of COVID-19-related knowledge, and the applicability of the research results to ordinary people is limited.

## Funding

This study was supported by Shaanxi Provincial Social Science Research Fund (No. 2020M016).

## Competing interests

None.

## Ethical approval

Not required.

## Authors’ contributions

GP and LJ are joint first authors. GP, LJ, TY, XX, and WY designed the study. GP, LJ, and TY collected the data. GP and LJ analyzed the data. GP, LJ, WS, HX, and TY drafted the manuscript. WS, WY, HX and XX contributed to the interpretation of the results and critical revision of the manuscript for important intellectual content and approved the final version of the manuscript. BY reviews and suggests the manuscript. All authors have read and approved the final manuscript. WY, GP, and LJ are the study guarantors.

## Data sharing

No additional data are available.
